# Screening of Discrete Wavelet Transform Parameters for the Denoising of Rolling Bearing Signals in Presence of Localised Defects

**DOI:** 10.3390/s23010008

**Published:** 2022-12-20

**Authors:** Eugenio Brusa, Cristiana Delprete, Simone Gargiuli, Lorenzo Giorio

**Affiliations:** Department of Mechanical and Aerospace Engineering (DIMEAS), Politecnico di Torino, 10129 Torino, Italy

**Keywords:** bearing fault diagnosis, discrete wavelet transform, thresholding, denoising, intelligent fault diagnosis, Case Western Reserve University

## Abstract

Maintenance scheduling is a fundamental element in industry, where excessive downtime can lead to considerable economic losses. Active monitoring systems of various components are ever more used, and rolling bearings can be identified as one of the primary causes of failure on production lines. Vibration signals extracted from bearings are affected by noise, which can make their nature unclear and the extraction and classification of features difficult. In recent years, the use of the discrete wavelet transform for denoising has been increasing, but studies in the literature that optimise all the parameters used in this process are lacking. In the current article, the authors present an algorithm to optimise the parameters required for denoising based on the discrete wavelet transform and thresholding. One-hundred sixty different configurations of the mother wavelet, threshold evaluation method, and threshold function are compared on the Case Western Reserve University database to obtain the best combination for bearing damage identification with an iterative method and are evaluated with tradeoff and kurtosis. The analysis results show that the best combination of parameters for denoising is dmey, rigrSURE, and the hard threshold. The signals were then distributed in a 2D plane for classification through an algorithm based on principal component analysis, which uses a preselection of features extracted in the time domain.

## 1. Introduction

The development of new technologies has promoted the application of some new smart techniques for machine control, enabling the transition driven by the Industry 4.0 strategic initiative. Bearings comprise one of the main components of rotating machinery, and their failure is often critical for system safety [1,2]. Detecting the presence of defects on a rolling bearing and predicting its residual life ensures an effective maintenance. This strategy is defined as Condition-Based Maintenance (CBM). The regular monitoring of system operation allows predicting more precisely the nucleation of defects in time and limiting the consequences of their occurrence, reducing the cases of catastrophic events and economic losses induced by machine stops.

Machine monitoring is often based on the analysis of vibrational signals extracted by sensors placed on or near the bearings. All extracted signals are subjected to a noise component, which inhibits the reading of useful monitoring information. The denoising process is often required and looks critical to ensure a proper extraction of data. The Wavelet Transform (WT) is one of the most-used techniques for denoising of signals [3,4]. The WT enables an analysis in the time–frequency domain, where the signal is compared with a family of functions called mother wavelets, which include parameters allowing the dilation or contraction of the frequency considered and a time window extension dependent on the frequency considered: low-frequency mother wavelets present a dilated time window, while high-frequency ones involve a smaller time window. Considering low-frequency mother wavelet are dilated with a large time steps, on the other hand, high frequencies involve smaller time steps and contracted mother wavelets. Numerous types of WTs are used in the literature for denoising processes such as the Continuous Wavelet Transform (CWT), Discrete Wavelet Transform (DWT), and Empirical Wavelet Transform (EWT). The goal of this paper is to design an algorithm for the screening of the denoising process, based on the DWT and applied to rotor bearing monitoring. The DWT is more used than the CWT because it gives enough information and a significant reduction in computational time [5]. Applying the DWT on a signal, two coefficient vectors are obtained, with two different frequency ranges, which describe the signal: the coefficient vector with a range in the higher frequencies is called the detail coefficient cD, while the one corresponding to lower frequencies is called the approximation coefficient cA. To further decompose the signal, the DWT is applied to the approximation coefficient cA just calculated.

In the literature, one of the most-common denoising methods that uses the DWT is based on thresholding the detail and approximation coefficients acquired by wavelet decomposition [2]. The concept behind this method is that the obtained coefficients contain information regarding a certain frequency band about the machines under observation. Wavelet coefficients contain both the target component of the signal and a noise component, which in many cases prevents the correct identification of the defect. The causes of noise are various, but when selective elimination is not possible, resorting to thresholding is suggested. In the literature, many authors use the WT for signal denoising for the extraction of fault features.

Many authors have studied the optimisation of parameters for denoising through the DWT usually focusing on a limited number of those. Some studies were performed to define the optimal decomposition level, as done by Sreejith et al. [6], who used the Morlet wavelet filters for the noise reduction of the vibrational signals from rolling bearings. Two algorithms were used for parameter optimisation before the denoising process. The algorithm that guaranteed the least computational time for process optimisation was based on using the Shannon entropy and kurtosis to optimise the decomposition level, which is therefore preferred for denoising the vibrational signals from rolling bearings. Sometimes, the optimisation of the decomposition level is followed by the selection of the best mother wavelet. Djebala et al. [7] focused on the analysis of the optimal level of wavelet decomposition. Then, using kurtosis as an optimisation parameter and considering only the Debauchies wavelet, they evaluated the most suitable mother wavelets as a function of the sampling frequency. In other cases, the decomposition level and mother wavelet are optimised together, as done by Sun et al. [8], who presented a new alternating current field measurement system and a wavelet-based noise reduction algorithm. They used a new evaluation parameter named Tradeoff (TO), presented in [9], and demonstrated that it was the best evaluation parameter compared to the others used.

The mother wavelet choice is one of the most important parts of the optimisation of the wavelet denoising, so there are some authors that considered only this as a variable parameter, as done by Rafiee et al. [10], who proposed an automatic feature extraction system for gear and rolling bearing diagnostics. They compared 324 wavelet families, evaluating the similarity of each of them with the analysed signals through a new evaluation algorithm. Secondly, they proposed a classification algorithm that compares the ranges of four statistical features using different mother wavelets. The results showed that the mother wavelet choice based on the signal was different from the one depending on the following process, such as denoising and classification. Another method to choose the optimal mother wavelet was presented by Kankar et al. [11], who carried out a study on feature extraction systems from rolling bearing signals by comparing seven mother wavelets. Shannon’s minimum entropy criterion was applied to all the signals, which were decomposed by the CWT with the seven different mother wavelets. The entropy was calculated for each wavelet coefficient and averaged over the whole signal. The wavelet family with the lowest sum of the averaged entropies was chosen. In wavelet denoising, a better optimisation is possible if the major parameterare considered. For example, Sadooghi and Khaidm [9] evaluated 84 combinations of 12 mother wavelets and seven threshold evaluation methods to optimise the noise reduction process using thresholding. They proposed a new evaluation parameter (Tradeoff (TO)) for identifying the best pair. Another approach to thresholding was presented by Li et al. [12], who discussed the different methods of signal segmentation for the application of the EWT, defining the adaptive one as the most-suitable method. The modes obtained from the EWT process were evaluated according to their energy content. In particular, the highest energy mode was subjected to the hard threshold, while all others were subjected to the implemented function, shown in the paper. The reliability of the EWT algorithm was guaranteed with a comparison with the Complete Ensemble Empirical Mode Decomposition (CEEMD), which demonstrated that the EWT process allows obtaining the best denoising of the signals. An example of the flexibility of the wavelet was presented by Wang et al. [13], who eliminated the noise component from the signals with a wavelet threshold denoising method. Therefore, the entropy value was extracted from the IMFs (Intrinsic Mode Functions), which were previously obtained from the EWT decomposition of the denoised signals.

The thresholding process is the most used in wavelet denoising, but there are different methods. Kedadouche et al. [14] introduced a new index to evaluate the wavelet coefficient based on the kurtosis. They calculated the kurtosis of every coefficient related to a faulty bearing with the same related to a healthy bearing and normalised it with respect to the difference of the kurtosis of the two signals considered. They evaluated that denoising using coefficients with a kurtosis index greater than one was better than using only the coefficient with the highest kurtosis index. In their work, a comparison between Empirical Mode Decomposition (EMD), ENSEMBLE Empirical Mode Decomposition (EEMD), and the EWT on the simulated signals was illustrated, and the results showed that the EWT denoising process was the best among the methods considered. Another approach to denoising was presented by Chegini et al. [2] based on the EWT, kurtosis, and envelope spectrum. The algorithm calculates, for each mode detected by the EWT, the so-called Pearson correlation coefficient and compares this value to that retrieved from experiments. Only when the value calculated is lower than the experimental one, the mode is discarded; otherwise, it is exploited to identify the occurring defect, through the thresholding process. Another denoising method using wavelets was analysed by Ge et al. [15], in which the IMFs were extracted from the signals by ensemble empirical mode decomposition, then the Pearson correlation coefficient was used to select the high-frequency IMFs; the high-frequency parts of the signals were denoised through the wavelet semi-soft threshold before reconstructing the signals, and the status of the rolling bearings was evaluated with the application of the multi-scale entropy method and support vector machine.

The WT is used for fault detection through the extraction of features from the wavelet coefficient, as done by Toma and Kim [16], who presented a rolling bearing failure classification scheme for induction motors using the motor electricity signal. The signal was subjected to the DWT for feature extraction; in particular, three wavelet families (Haar, db4, and sym4) were compared by decomposing each signal to the eleventh level. The matrix obtained was used as the input for machine learning ensemble classifiers. The aim of the paper was to achieve high classification accuracy by reducing the computational power required. Another approach was presented by Ziani et al. [17], who proposed an approach of fault detection based on three steps: feature extraction, selection of sensitive features, and classification. Feature extraction took place after the signal was decomposed through Wavelet Packet Decomposition (WPD). Both the time and frequency domain characteristics of the signal were picked out to optimise the classification. The selection method consisted of scoring, according to three different criteria, each of the features considered, then, after sorting the features in descending order of score, the values of the first features were added together to reach 80% of the total score, and these features were the ones used in the classification method. In the literature, a multitude of different techniques are presented for classifying signals such as the one reported by Li et al. [18], who, in order to study the complexity of ship-radiated noise, introduced the use of the Slope Entropy (SloE) in underwater acoustic signal processing. They suggested a Variational Mode Decomposition (VMD) and SloE-based feature extraction technique for ship-radiated noise. They came to the conclusion that the method utilising the SloE had the highest recognition rate while considering one characteristic or more, with the same amount of features taken into account, after comparing this method with other techniques used. Another approach to denoising was developed by Zhou et al. [19], which combines the fuzzy entropy discriminant as the threshold with Empirical Ensemble Mode Decomposition (EEMD) and Independent Component Analysis (ICA). In order to achieve end-to-end fault diagnosis, they also created an improved Convolutional Neural Network (CNN) model. The experimental findings showed the high accuracy and excellent anti-interference capacity, but with limitations such as the designed model’s real-time performance.

Many publications are present in the literature focusing on denoising optimisation with the DWT, but there are no papers where all parameters are optimised. In some cases, only the optimal mother wavelet is evaluated, basing the choice on the similarity of the wavelet to the signal [10]. In others, very few mother wavelets and just some levels of decomposition are compared to evaluate which is the best pair [8]. Studies are also available in the literature where the best pair between the mother wavelet and threshold technique is assessed [9], or only the best function for applying the calculated threshold value is evaluated [2].

Although research on the use of machine learning (ML) techniques for rotating systems’ monitoring has been expanding in recent years, the industry sector still remains interested in employing monitoring techniques based on known algorithms that can provide a more extensive level of knowledge insight. In this context, denoising algorithms based on the DWT still receive high interest, due to their good behaviour, as verified in the literature. However, there still remain some research gaps regarding the screening of the complete set of parameters involved in this technique. Comparative studies considering all parameters that may vary in the DWT, specifically the mother wavelet, threshold evaluation, and threshold function, are not available. The main goal of the present paper is to select the best combination of the decomposition level, mother wavelet, thresholding rule, and thresholding function for the denoising of the vibration signal of rolling bearings, which cannot be found in the literature. A fundamental assumption of this approach is that the most-effective denoising optimisation is reached when the highest number of system parameters is simultaneously considered. Thus, the decomposition level is calculated considering the type of signal and the rolling bearing under study, while the mother wavelet, threshold value, and threshold function are chosen through iterations of the algorithm. For this research work, 10 vibration signals related to the Case Western Reserve University (CWRU) database were denoised with 160 different groups of parameters. Every case was evaluated from two different features, i.e., kurtosis and Tradeoff (TO). At the end, two groups of parameters were extracted that optimised the kurtosis and TO. Comparing the results of these two groups, it is possible to define the best parameters for the signals analysed. After that, 108 signals from the CWRU database were denoised using the parameters just found, and with the denoised signal, the classification algorithm was trained. The aim of the algorithm is to determine the nature of the vibrational signal derived from the rolling bearing using only features extracted from the time domain.

The screening activity of the DWT parameters performed in the present paper will be extended in future works by including signals from different test benches to achieve the optimal parameters for more generalised sets of bearing signals; in particular, the proposed algorithm will be applied to signals extracted from an innovative test rig for industrial bearing monitoring presented in [20].

## 2. Materials and Methods

**Figure 1:**
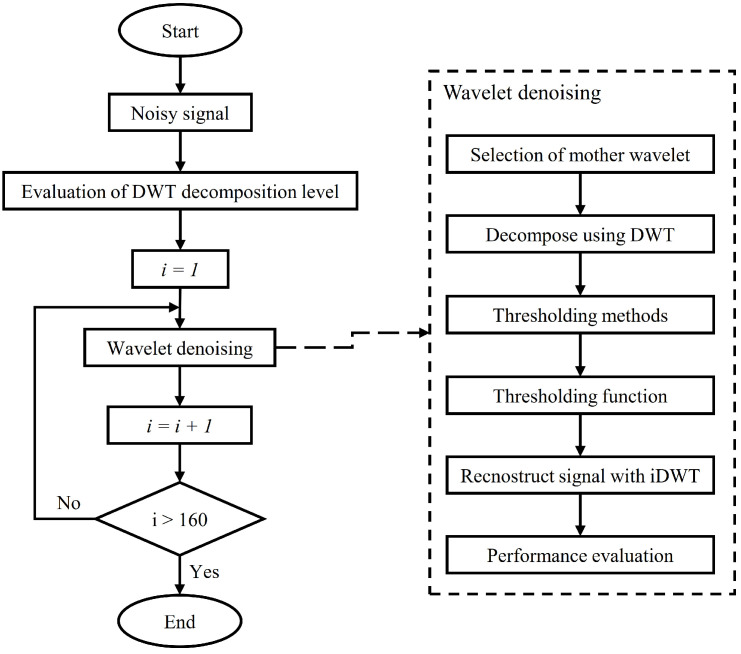
Algorithm flow chart for the parameter screening of the proposed DWT denoising.

In this paper, a screening algorithm is proposed to obtain the best wavelet parameters for wavelet denoising. The proposed algorithm, presented in Figure 1, uses the signal of the CWRU database of healthy and faulty rolling bearings to find the best combination of the decomposition level, mother wavelet, threshold value, and threshold function. A thorough explanation of each step in the screening algorithm is presented in the following.

In Figure 1, the various steps of the parameter screening algorithm applied to each analysed signal are reported. The first step is to calculate the level of decomposition known from the signal information and the type of rolling bearings studied. Then, through an iterative process, the 160 denoising methods, related to the combination of 10 mother wavelets, five threshold rules, and three threshold functions and using the index kurtosis technique [14] with all the mother wavelet were compared. Each combination was evaluated using two criteria. This system has to be applied to several signals from the same database to identify the best combination.

The next step is the training of a signal classification system that consists of three steps: the evaluation of the features by the Fisher score [21], the selection of relevant features according to the Pareto principle [22], and finally, if necessary, the extraction of the principal components using Principal Component Analysis (PCA) [23].

### 2.1. Calculation of the Decomposition Level

Typically, in the literature [2,7,24], the characteristic frequency and its harmonics are used to detect the presence of defects on rolling bearings. According to Djebala et al. [7], at least three harmonics have to be highlighted to facilitate defect identification. This means that the last frequency band relative to the approximation coefficient must contain at least three harmonics of the characteristic frequency of the signal.

**Figure 2:**
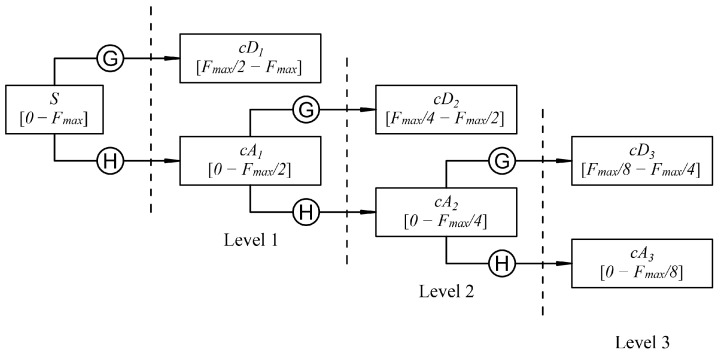
Identification of frequency bands related to the various coefficients as a function of the decomposition level. G indicates the high-pass filter and the following down sampling, while H indicates the low pass filter and the following down sampling.

The algorithm for the evaluation of the detail and approximation coefficients as a function of the decomposition level is shown in Figure 2, where *S* is the signal, $cD_{n}$ is the detail coefficient, $cA_{n}$ is the approximation coefficient, and $F_{max}$ is the maximum frequency of *S*.

To acquire the detail and approximation coefficients of the first decomposition level, the signal is processed through a high-pass and low-pass filter, followed by down sampling. When $F_{max}$ is the signal maximum frequency, the approximation coefficient frequency band is 0 to $F_{max}/2$, while the detail coefficient frequency band is $F_{max}/2$ to $F_{max}$. For decomposition levels after the first, the same procedure is applied on the approximation coefficient just calculated, to obtain the detail and approximation coefficients of the next level. The frequency bands of the new coefficients are half of the approximations’ coefficient band. Considering a decomposition level *n*, at the end of the decomposition process, the last approximation coefficient and the *n* detail coefficients calculated are considered for the post-processing operations. Therefore, the maximum frequency of the last level approximation coefficient can be calculated as:

(1) $$F_{max}\left(cA_{n}\right)=\frac{F_{max}\left(S\right)}{2^{n}}$$

where *S* is the signal, $cA_{n}$ is the approximation coefficient, $F_{max}\left(\right)$ indicates the maximum frequency of the input, and *n* is the decomposition level.

Considering as the upper limit the maximum frequency of the last level approximation coefficient halfway between the third and fourth harmonics, the decomposition level can therefore be calculated inverting (1) as:

(2) $$n=1.44\cdot \log_{10}\left(\frac{F_{max}\left(S\right)}{3.5\cdot F_{c}}\right)$$

where $F_{c}$ is the characteristic frequency related to the bearing defect.

The bearing characteristic frequencies result from the rolling elements impacting a localised defect on the raceways or cage of the bearing itself or from the interaction between a defect on one or more rolling elements and another rolling bearing component. The contact generates an acceleration impulse in the sensor signals, which becomes also visible in the frequency spectrum of the signal. The characteristic frequencies of rolling bearings are:

- Ball Pass Frequency Outer raceway (BPFO), i.e., the frequency of the passage of a rolling element at the same point on the outer raceway;
- Ball Pass Frequency Inner raceway (BPFI), i.e., the frequency of the passage of a rolling element at the same point on the inner raceway;
- Ball Spin Frequency (BSF), i.e., the frequency of rotation of the rolling elements around their axis, since statistically, the defect on the rolling element impacts a rotation both on the inner and outer raceway, in the frequency spectrum, and there will be peaks in 2×BSF and its harmonics;
- Fundamental Train Frequency (FTF), i.e., fundamental frequency of rotation that corresponds to the frequency of rotation of the cage.

If the nature of the defect is known, the decomposition level for calculatingthe characteristic frequency considered will be the corresponding one. Otherwise, the maximum among the characteristic frequencies of the considered rolling bearings is taken as the reference.

### 2.2. Choice of Wavelet Family

Although literature studies are present that link the choice of the wavelet family only to the analysed signal [16,25], it has become clear in the latest studies that the choice should also depend on the thresholding method applied to the wavelet coefficients and the evaluation parameter used to define the best denoising method [10]. The most-used wavelet families were selected from the literature, from which it is possible to derive mother wavelets by means of coefficients that modify their structure. Table 1 lists the mother wavelets used in this paper and the literature sources from which they were derived.

### 2.3. Identification of Thresholding Rule

In this section, the five thresholding rules used as the parameters of the denoising screening algorithm are described. The thresholding process was first presented by Donoho and Johnstone [26] in 1994. The idea is to compare each element of the vectors of detail and approximation coefficients obtained from the DWT with a threshold value. Different thresholding methods have been presented in the literature, and five types of threshold value techniques were used in the present study to define the best one for the denoising of rolling bearing signals: universal threshold, rigorous Stein’s Unbiased Risk Estimate (rigrSURE), heuristic SURE (heuresure), minimax, and penalised method.

The universal threshold evaluates only one threshold value for every DWT coefficient [27], based on the length of the signal analysed. This method saves computational time, but does not consider the real noise distribution in each coefficient.

rigrSURE is an adaptive thresholding method based on Stein’s unbiased likelihood estimation principle, which was introduced by Donoho and Jonstone [28]. The expression and demonstration of such a method are shown in [28] and consist of a likelihood estimation using a given threshold th, then minimising the non-similarity with th to obtain the threshold value. Indeed, in large dimensions, the vector will be set to a sort of statistical regularity, while will ensure the close proximity between SURE and the true risk, and that $th_{rigrSURE}$ will be nearly the best threshold. According to [28], this method has a computational cost a little bit greater than others.

The heuresure threshold is a combination of rigrSURE and the universal threshold method [9,29]. The SURE method would be unreliable if the Signal-to-Noise Ratio (SNR) is very small, while the universal threshold gives a better threshold assessment. If the SNR is higher, the rigrSURE threshold method is used instead.

The minimax method uses the minimax principle to find a threshold for every coefficient, thus the denoising of signals can be seen similar to the estimation of an unknown regression function. minimax allows the evaluation of the function that minimises the maximum value of the Mean-Squared Error (MSE) among the proposed functions [9].

The penalised method, also referred to as the Birgé–Massart method, is the best-known technique for calculating the threshold value among the parametric methods. In this threshold method, the detail and approximation coefficients are sorted in descending order of their absolute value, after which the threshold value is calculated [9,30]. The expression is regulated by a penalised parameter ($\alpha_{p}$), which gives the ability to identify three different intervals [9]:

- Penalised high, $2.5<\alpha_{p}<10$;
- Penalised medium, $1.5<\alpha_{p}<2.5$;
- Penalised low, $1<\alpha_{p}<2$.

**Figure 3:**
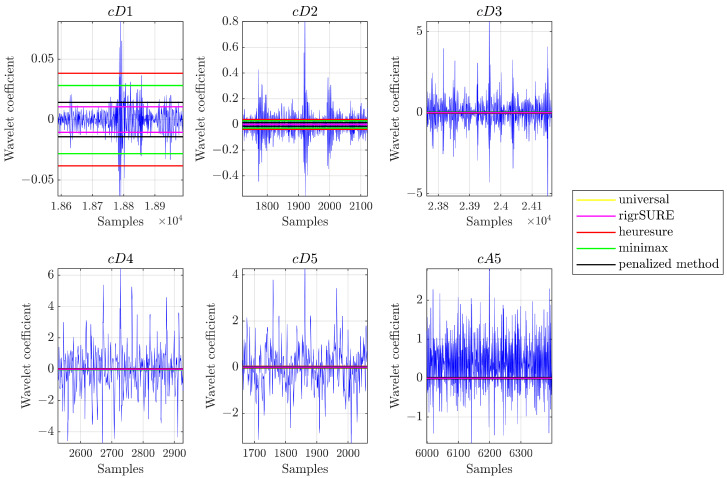
Example of threshold application on wavelet coefficients of signals from rolling bearings.

Threshold determination is an important question in the denoising process. A small threshold may yield a result that may be noisy, and a large threshold can cut a significant part of the signal, thus losing the important details of the signal [31]. Figure 3 allows seeing the threshold values calculated by the different methods described for a signal relating to a rolling bearing (SKF 6205-2RS JEM) with a defect on the inner raceway with a diameter of 0.007 in. The universal threshold and minimax provide a unique threshold value for each level; this is true for the heuresure method also, which in this case provides a threshold value equal to the universal threshold method, as the analysed signal presents a small SNR value.

### 2.4. Thresholding Function

Various methodologies can be used to apply the evaluated threshold value, the most common of which are the hard threshold and soft threshold; there are, however, many functions that allow having a middle way between these two types. The hard threshold function generates a vector of the same dimension of the input, in which the absolute value of each element is compared with the threshold value th. If the absolute value results in being lower than the threshold, it would be set equal to zero in the output vector; otherwise, it can be considered equal to the value of the original signal. Due to the discontinuities that are created, this method causes fluctuations in the signal after reconstruction, even though the local properties of the raw signal are preserved. Thus, the equation for the general *k*-th wavelet coefficient $c_{(}i,k)$ at the decomposition level *i* is [2,9,12]:

(3) $$c_{i,k}^{th}=\left\{\begin{array}{cc} c_{i,k} & \left|c_{i,k}\right|\geq th_{i}\\ 0 & \left|c_{i,k}\right|<th_{i} \end{array}\right.$$

The soft threshold function differs from the previous one because the retained values are centred on the threshold. After thresholding, a high-frequency part of the coefficients is lost, and they are still consistent. In this case, the equation for evaluating the wavelet coefficient of the output vector becomes [2,9,12]:

(4) $$c_{i,k}^{th}=\left\{\begin{array}{cc} \mathrm{sgn}\left(c_{i,k}\right)\left(\left|c_{i,k}\right|-th\right) & \left|c_{i,k}\right|\geq th_{i}\\ 0 & \left|c_{i,k}\right|<th_{i} \end{array}\right.$$

Li et al. [12] implemented a new function that allows overcoming the disadvantages of the two previous method, as follows:

(5) $$c_{i,k}^{th}=\left\{\begin{array}{cc} \left(1-\mu \right)c_{i,k}+\mu \cdot \mathrm{sgn}\left(c_{i,k}\right)\left(\left|c_{i,k}\right|-th_{i}\right) & \left|c_{i,k}\right|\geq th_{i}\\ 0 & \left|c_{i,k}\right|<th_{i} \end{array}\right.$$

with:

(6) $$\mu =\alpha^{{\left(\left|c_{i,k}\right|-th_{i}\right)}^{2}}\;\mathrm{with}\;0\leq \alpha \leq 1\Rightarrow 0\leq \mu \leq 1$$

The choice of the alpha coefficient allows the selection of different functions within the extremes of the hard and soft thresholds, in particular for:

- α=0, the hard threshold function is obtained;
- α=1, the soft threshold function is obtained;
- 0<α<1, continuous threshold functions are obtained, which for a value near th are similar to the soft threshold and for a value far from th are similar to the hard threshold.

**Figure 4:**
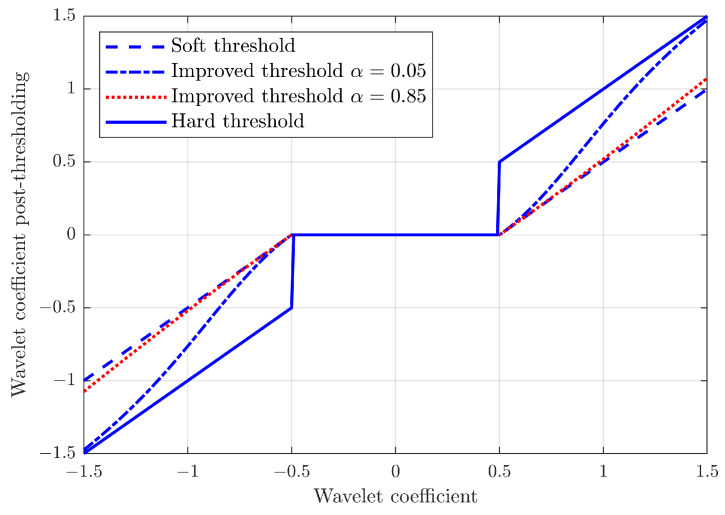
Development of different threshold functions, with a threshold value of th=0.5.

Figure 4 describes the improved threshold applied to a coefficient characterised by the function y=x. As can be observed, an increase in the α coefficient implies a reduction of the coefficients also outside of the threshold region, resulting in a lower amplitude of the denoised signal compared to the raw one. In order to limit this effect, a low value of α may be preferable, and in the current study, a value of α=0.05 was chosen for the improved threshold function [12].

The hard threshold and improved threshold were selected as the first two threshold functions to be compared in the screening algorithm presented. The third threshold function used, denoted as the mixed threshold, is a combination of the hard threshold and improved threshold function. In particular, the coefficient with the maximum energy was analysed with the hard threshold, whereas all other coefficients were analysed with the improved threshold function, as shown in [12].

Changing the approach, instead of selecting each element of the coefficients individually, the coefficients with the most general information can be selected. This method is based on the fact that some coefficients are closely correlated with bearing faults, while others contain information that is unnecessary for such diagnostics, including noise. Kedadouche et al. [14] presented a new method for identifying the coefficients most closely related to the presence or absence of a defect based on the kurtosis. This is possible because healthy bearings present a kurtosis close to 3, while in the case of damage, the kurtosis tends to assume higher values. The method considers the difference between the kurtosis of coefficients corresponding to the same frequency range for a damaged and a healthy bearing for all wavelet coefficients. This value is normalised to the difference between the kurtosis of the raw signal under test and a healthy bearing signal. The frequency ranges of the wavelet coefficients are the same for both the signal under test and the healthy bearing signal if they are taken from bearings of the same model and with the same sampling frequency. The equation of this new method is:

(7) $$kurtosis\_index_{i}=\frac{\mathrm{kurtosis}{\left(c_{i}\right)}_{\mathrm{damaged}}-\mathrm{kurtosis}{\left(c_{i}\right)}_{\mathrm{healty}}}{\mathrm{kurtosis}{\left(x\right)}_{\mathrm{damaged}}-\mathrm{kurtosis}{\left(x\right)}_{\mathrm{healthy}}}$$

where $c_{i}$ denotes the wavelet coefficient of the *i*-th decomposition level and *x* denotes the raw signal in the defective or healthy case.

All coefficients with a kurtosis index greater than or equal to one are used to reconstruct the signal for indirect signal denoising. This approach can only be applied if there is a history of signals related to the rolling bearings under consideration or signals related to the same bearings when these signals can be defined as healthy. Therefore, it cannot be applied as a control system for existing and already operating configurations.

### 2.5. Performance Evaluation

In the present paper, each signal was subjected to 160 different combinations of denoising, so the choice of the evaluation parameters is crucial. The two parameters chosen for performance evaluation were the kurtosis ($k_{1}$) and Tradeoff (TO).

The first allows identifying the presence or absence of a defect in the rolling bearings. The kurtosis corresponds to the fourth centred moment, normalised by dividing it by the fourth power of the standard deviation, and represents a statistical measure used to describe the distribution of observed data around the mean. The kurtosis presents a value of 3 if the distribution is normal (Gaussian); if it is larger than 3, it corresponds to a more spiky distribution, whereas if the kurtosis value is less than 3, there is a flatter distribution. In this paper, the kurtosis was calculated according to the formulation:

(8) $$k_{1}=\frac{\frac{1}{n}\sum_{j=1}^{n}{\left(x_{j}-\bar{x}\right)}^{4}}{{\left[\frac{1}{n}\sum_{j=1}^{n}{\left(x_{j}-\bar{x}\right)}^{2}\right]}^{2}}$$

where *n* is the number of elements of the signal, $x_{j}$ is the *j*-th element of the signal, and $\bar{x}$ is the mean.

Studying signals from rolling bearings, the kurtosis takes on values of 3 in the case of a healthy bearing. On the other hand, the kurtosis for damaged bearings assumes values greater than 3. In addition, screening of this parameter provides indirect screening of the denoising of the signal. Other parameters that are used in the literature to highlight the presence or not of defects, and become more effective when the size of the defect is considerable, are the Peak Value (PV) or the Root Mean Square (RMS) and all the characteristics derived from them.

The second parameter is a mix of features that directly assess the denoising of the signal by comparing the post-processed signal with the raw signal or the extracted noise. This was presented by Sadooghi and Khadem [9]. The formulation used is [32]:

(9) $$\mathrm{TO}=\frac{\mathrm{SNR}\cdot xcorr}{\mathrm{PrmsD}}$$

where SNR is the Signal-to-Noise Ratio, xcorr is the cross-correlation factor, for which large values are preferred, and PrmsD is the Percentage root-mean-squared Difference for which small values are desirable.

Sun et al. [8] demonstrated the effectiveness of TO by comparing the denoising results obtained from the combinations that maximise the TO parameter with those that achieve the maximum SNR value, the maximum xcorr value, and the minimum PrmsD value. They showed that the denoising obtained by the combination that maximises TO is better than the others and that the denoising obtained by the maximum value of xcorr is close to the best.

### 2.6. Classification Algorithm

Classification is a fundamental step in machine diagnostics. This paper is focused on identifying the nature of the defect by pre-selecting features and applying Principal Component Analysis (PCA) to obtain a graphical distribution of the signals.

The features’ pre-selection is based on the scheme presented in [17], with the evaluation carried out by means of the Fisher score. The idea behind the method is that the distance between points of different classes is the maximum possible, while the distance between points of the same class is the minimum possible.

The features’ evaluation permits defining a ranking that will allow the application of the Pareto method for selection. This is a technique for decision-making based on the Pareto principle, also known as the 80/20 rule [22]. The analysis is based on the idea that 80% of the benefit can be achieved by doing 20% of the work [17]. The objective of this paper was to create a subset of features to be provided as the input to the PCA in order to obtain the classification of the analysed signals. Thus, the selected features are those that allow obtaining 80% of the evaluation metric used. The features’ pre-selection permits improving the results obtained from the application of the PCA.

PCA is a technique that uses singular-value decomposition to obtain a hierarchical coordinate system for representing data. Principal Components (PCs) represent the new coordinate system that maximises the correlation with measurements. This technique can be seen as an eigenvalue problem.

The eigenvalues indicate the variance of the principal component of the data, thus indicating the importance of a component. This technique allows condensing a considerable amount of information about the same signal into fewer components, allowing identifying the state of each signal and distinguishing them from the others.

The aim of this algorithm is to show a first approach to the classification of the signals under investigation.

## 3. Evaluation of Denoising Screening Results

Applying the screening algorithm to signals from the CWRU database [33,34] reveals the best technique for denoising using the DWT. In this section, the combinations that optimise the evaluation parameters is discussed and compared with each other. In Table A1 in the Appendix A, the variables corresponding to each index are shown.

The signals of the CWRU database were obtained from a test stand composed of a 2 hp Reliance Electric motor and two bearing localised near to and far from the motor. The vibration data were collected using accelerometers, which were attached to the bearing housing. The signals were extracted from SKF rolling bearings 6205-2RS JEM with a single-point fault for bearings with diameters of 0.007 in, 0.014 in, and 0.021 in. The vibration signals were collected with a sampling frequency of 12 kHz for each cases and 48 kHz for the drive end bearing faults. The faulty bearings on the outer raceway were assembled with the damage at 3 o’clock (orthogonal to the load zone), at 6 o’clock (in the load zone), and at 12 o’clock (in opposition to the load zone).

**Figure 5:**
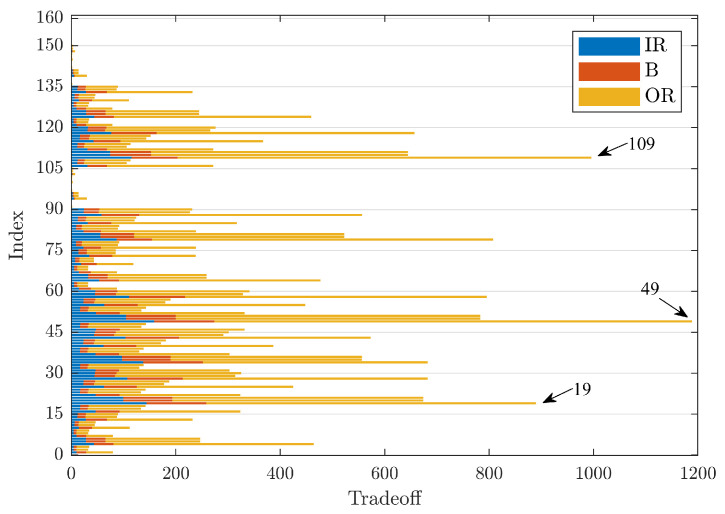
Comparison of TO values for defects of size 0.007 in.

The first evaluation focused on the denoising efficiency for signals from faulty bearings with a size of 0.007 in and a rotation speed of 1797 rpm, considering the three defect positions between the main elements of a rolling bearing. Figure 5 is a bar graph, where the y-axis shows the indices used for the identification of the denoising technique under investigation; the TO value is on the x-axis. Each column is the sum of the TO values obtained after denoising from bearings with defects localised on the inner raceway (IR, in blue), on the rolling element (B, in red), and on the outer raceway (OR, in yellow), with the same diameter.

Figure 5 shows that Index 49 has the highest sum of TO values (TO=1189) followed by Indices 109 and 19 with a TO value of 996 and 889, respectively. These indices have the same technique for threshold evaluation and the same threshold function, respectively, rigrSURE and hard threshold, but with three different mother wavelets: dmey, coif5, and db44.

Another observation is that the index intervals 91 ÷ 105, 136 ÷ 150 present very low TO values, a sign that the corresponding mother wavelets, respectively rbior1.1 and haar, are not suitable for the use of these techniques. Moreover, it can be noted that the denoising carried out by the application of the kurtosis indicator, corresponding to the indices 151 ÷ 160, is not very effective, as they produce lower TO values. Furthermore, it can be seen that the highest TO value in each individual column is given by the OR signals followed by IR and, finally, by B.

**Figure 6:**
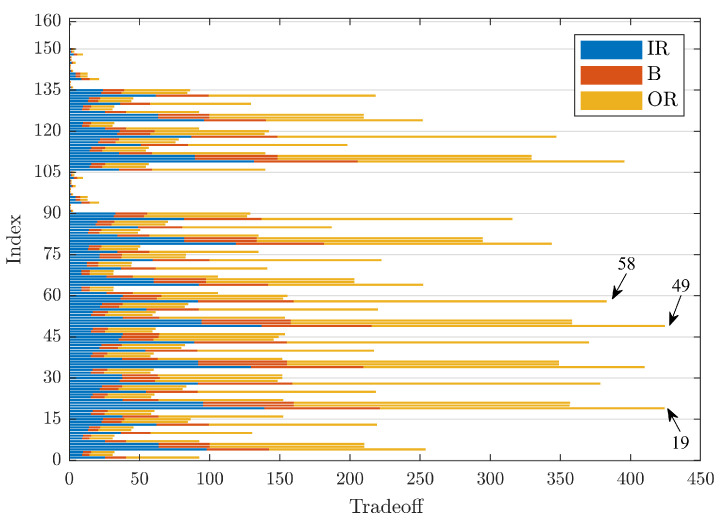
Comparison of TO values for defects of size 0.014 in.

Turning to the case of a defect with a diameter of 0.014 in, Figure 6 shows the TO trend, as in Figure 5. The indices that guarantee the highest TO values are, in order, 49 and 19, respectively, with a TO value of 425 and 424. The values obtained are about 1/3 of those obtained in the previous case. As in the previous case, there are mother wavelets with very low TO values, namely Indices 91 ÷ 105, which correspond to rbior1.1, and Indices 136 ÷ 150, which correspond to haar. Moreover, also in this case, the indices related to the kurtosis indicator method are not optimised for TO.

In this case also, the group of variables with index 49 is the best for tradeoff, but from Table 2, it can be noted that the maximum for each type of defect is obtained by Indices 19 (for IR and B signals) and 58 (for OR signals).

**Figure 7:**
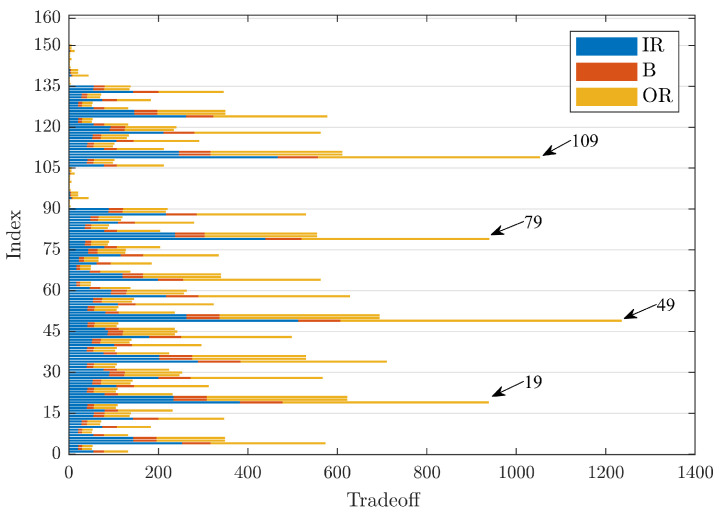
Comparison of TO values for defects of size 0.021 in.

Finally, analysing the signals for the rolling elements’ bearings with a defect diameter of 0.021 in, Figure 7 shows the TO values. In this case, it can be seen that the order of magnitude of the maximum values again stabilised at values comparable with the case of a defect with a diameter of 0.007 in, although the TO values for the IR signals are comparable with those obtained for the OR signals. The maximum TO value is 1236, and it was obtained for Index 49, followed, in order, by Indices 109, 79, and 19, with values of 1053, 940, and 939, respectively. These four indices stand out among the others. Thresholding was applied through rigrSURE and the hard threshold, while the mother wavelets changed and corresponded, respectively, to dmey, coif5, bior6.8, and db44.

Furthermore, in this case, the mother wavelets rbior1.1 and haar and the kurtosis indicator were unsuitable if TO was used as a denoising metric, making these mother wavelets not suitable for denoising the analysed signals.

Table 3 summarises the results shown in Figure 5, Figure 6 and Figure 7, listing the denoising parameters that maximise the sum of the TO values at varying defect diameters. It was confirmed that Index 49 clearly indicates the best group of parameters that maximise the sum of the TO values.

**Figure 8:**
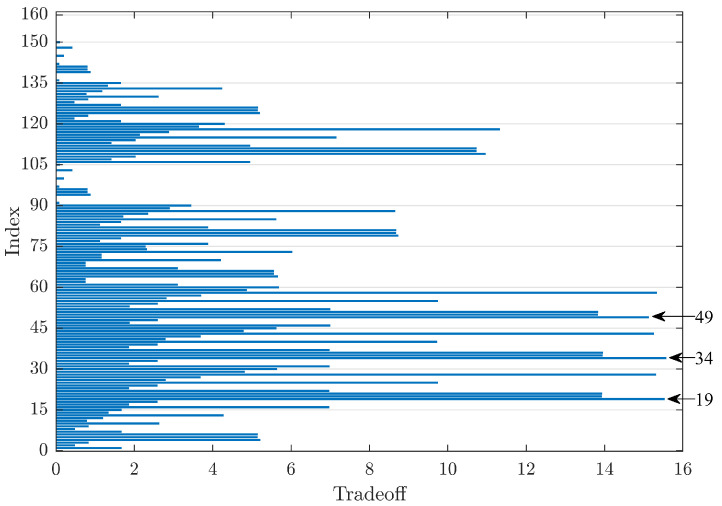
Comparison of TO values for healthy bearings.

The evaluation of the denoising obtained by the signal from a healthy bearing (indicated as N) allows estimating all the main case histories in the use of a rolling element bearing in industry. Figure 8 shows such a trend and highlights peaks corresponding to rigrSURE and the hard threshold as the methods for noise reduction. The wavelet families that guarantee higher TO values are, in order: db45, db44, and dmey. In this case, Index 49 provides the third-highest value of TO, with values very close to the first, Index 34, with a difference of only 0.45 points. Finally, the graph shows that, for signals from healthy bearings, the implemented threshold function and the mixed one guarantee almost identical values.

The evaluation of the best group of parameters that gives the maximum TO allowed deriving the results shown in Table 4. dmey and rigrSURE are, respectively, the mother wavelet and threshold rule that optimise most signals, while the hard threshold is the best technique among those considered for all the signals analysed. Thus, Index 49 identifies the best group of parameters for wavelet denoising for the signals under consideration.

Sadooghi and Khaidm [9], using signals from a cargo plane jet engine, tried to define the best combination of the mother wavelet and threshold evaluation method using TO as the evaluation parameter. The results showed in [9] highlight dmey and rigrSURE as the best parameters for the denoising process, in accord with the results illustrated in this work; furthermore, also in their case, rigrSURE allowed obtaining the first three highest TO values. In their study, the mother wavelets with the second and third TO value were, in order, sym8 and bior6.8, with a 25 percent decrease in the TO value for each; in the present study, the second and third mother wavelets that maximise TO are, in order, coif5 and db44, with a TO value reduction of 12 and 18 percent, respectively; this difference in the second and third mother wavelets can be associated with the different nature of the signal used, which consequently resulted in signals more similar to particular mother wavelets than others. In the current research, using signals from rolling bearings with localised defects, the sym family did not result in a high TO value, while the bior6.8 mother wavelet (considering Index 79 with the rigrSURE threshold evaluation method and hard threshold function) was one of the parameter sets with a higher TO value, up to the fourth highest in the case of defects with sizes of 0.007 in and 0.021 in.

Observing the trend of the kurtosis values obtained from the various analyses, the indices that made it possible to assess the presence or absence of a defect on the rolling bearings as best as possible were obtained. For signals from faulty bearings, the goal is to maximise the kurtosis value to highlight the presence of a defect.

**Figure 9:**
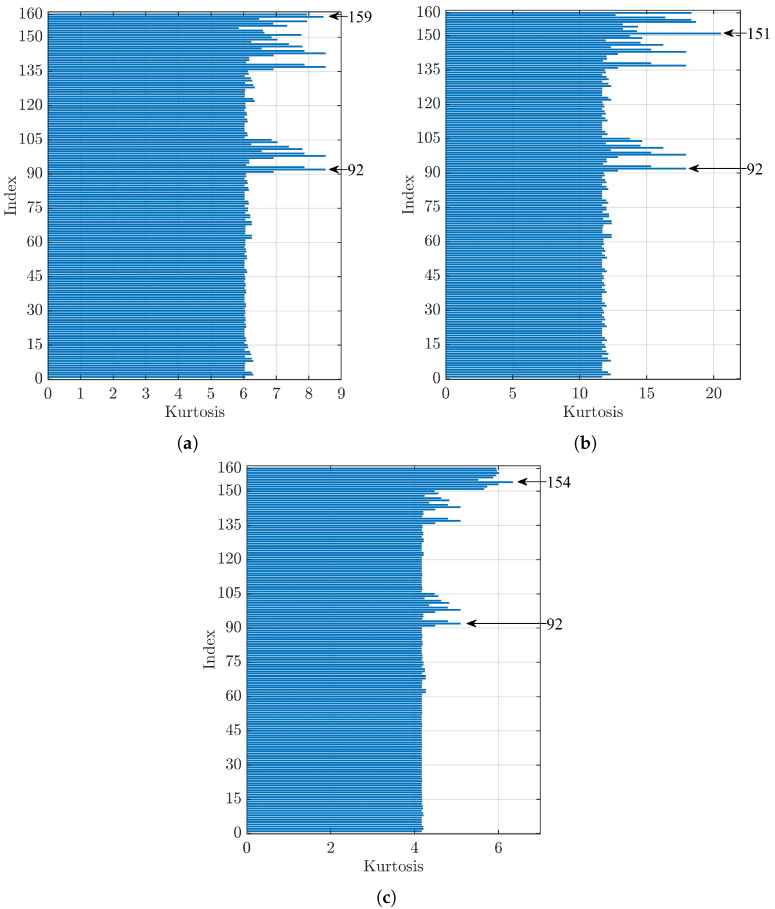
Comparison of kurtosis values for a bearing with a defect on the inner ring (IR) of diameter: (**a**) 0.007 in, (**b**) 0.014 in, and (**c**) 0.021 in.

Figure 9 shows the values obtained by analysing the signal from bearings with defects on the inner ring with a diameter of 0.007 in (a), 0.014 in (b), and 0.021 in (c). In Figure 9a, Index 92 has the highest kurtosis value, Index 159, corresponding to sym4 as the mother wavelet and the kurtosis index as the denoising method, is the first in the group using the kurtosis index (Indices 151–160) and presents the fifth-highest kurtosis value, with a difference of 0.8% compared to Index 92. Figure 9b shows that Index 151 (mother wavelet db4 and kurtosis index) has the highest kurtosis value, while Index 92 is in fifth place with a difference of 12.7% with respect to the first one. For Figure 9c, between the indices that used the kurtosis index as the denoising method, the maximum kurtosis value was reached by Index 154 (dmey as the mother wavelet), while considering all parameter sets that used the threshold method (Indices 1–150), Index 92 gave the highest kurtosis value, with a difference of 19.7% with respect to Index 154.

**Figure 10:**
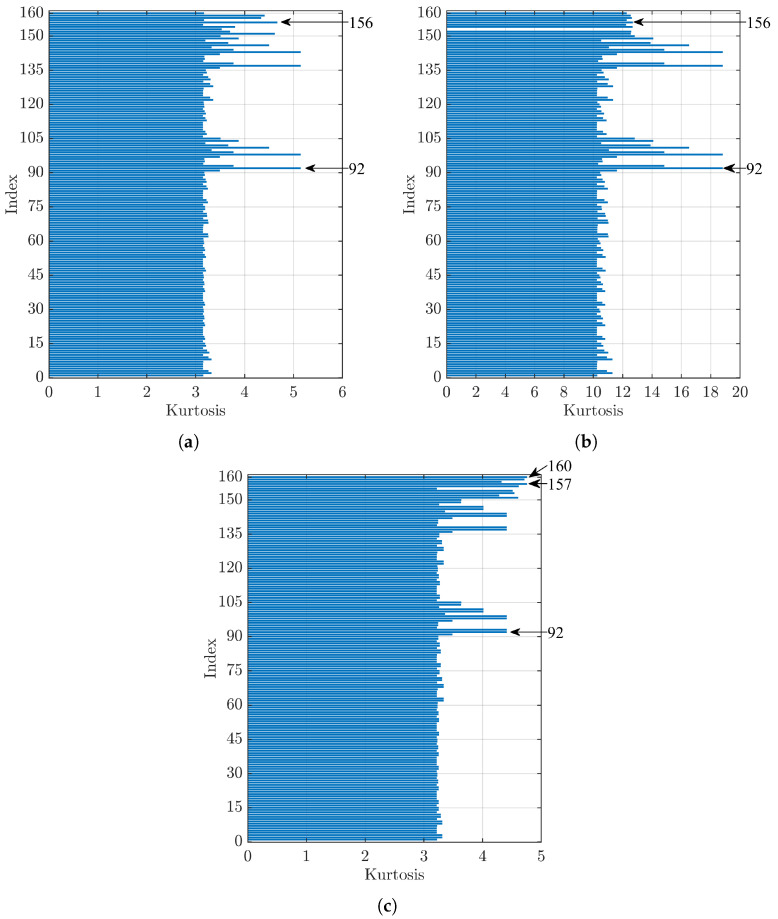
Comparison of kurtosis values for a bearing with a defect on the rolling element (B) of diameter: (**a**) 0.007 in, (**b**) 0.014 in, and (**c**) 0.021 in.

Figure 10 shows the kurtosis index trends for signals related to rolling bearings with defects on the roller element with a diameter of 0.007 in (a), 0.014 in (b), and 0.021 in (c). Figure 10a,b shows that Index 92 maximised the kurtosis value, while the first index that used the kurtosis index as the denoising method, which gave the maximum value, was 156 corresponding to the bior6.8 mother wavelet, respectively, in fifth place with a difference of 9.3% and in seventeenth place with a difference of 32.6% with respect to the related first one. On the other hand, Figure 10c shows that, for the highest dimension of defect on the rolling element, the first two indices that maximised the kurtosis value were 157 and 160, corresponding to the mother wavelets rbior1.1 and haar, respectively. In this case, the index with the highest value that uses the threshold method was 92, with a difference of 7.3% with respect to Index 157.

**Figure 11:**
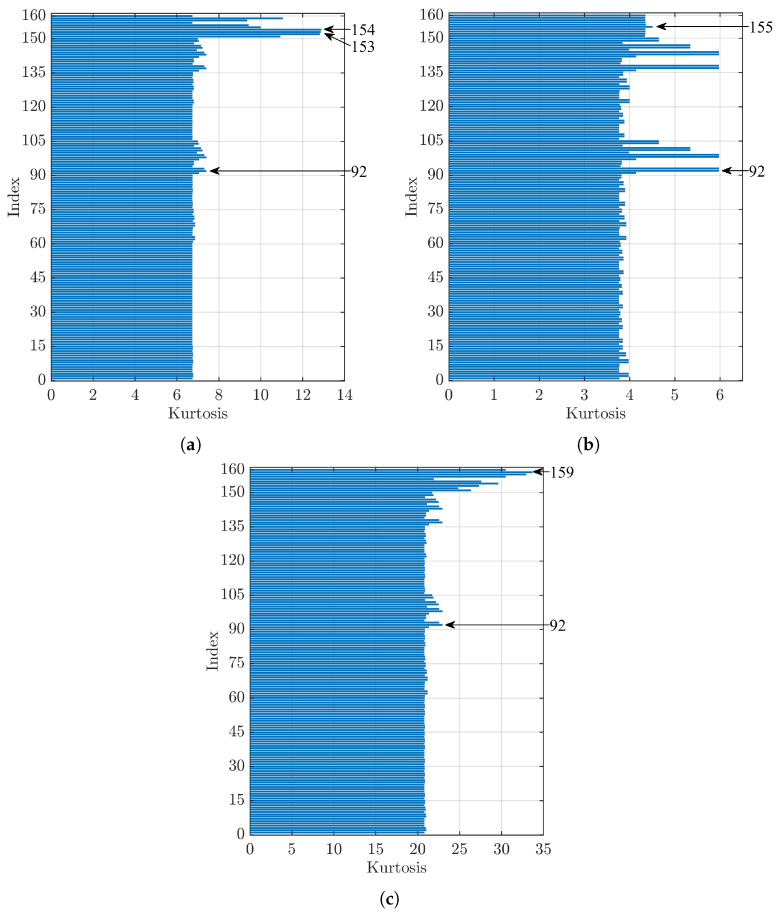
Comparison of kurtosis values for a bearing with a defect on the outer ring (OR) of diameter: (**a**) 0.007 in, (**b**) 0.014 in, and (**c**) 0.021 in.

Figure 11 shows the values obtained by analysing the signal from bearings with a localised defect on the outer ring with a diameter of 0.007 in (a), 0.014 in (b), and 0.021 in (c). In Figure 11a, Indices 154 (dmey) and 153 (db45) have the first two highest kurtosis values, while Index 92, which is the index with the highest value that used the threshold as the denoising method, presents a difference of 42.5% with respect to Index 154. Figure 11b shows that Index 92 has the highest kurtosis value, while Index 155 presents the highest value between the sets that employed the kurtosis index method and a difference of 24.6% with respect to the first one. For Figure 11c, Index 159 (sym4) maximises the kurtosis value, while Index 92, with a difference of 31.9% with respect to Index 159, presents the highest kurtosis value in the group of parameter sets that employed the threshold method.

**Figure 12:**
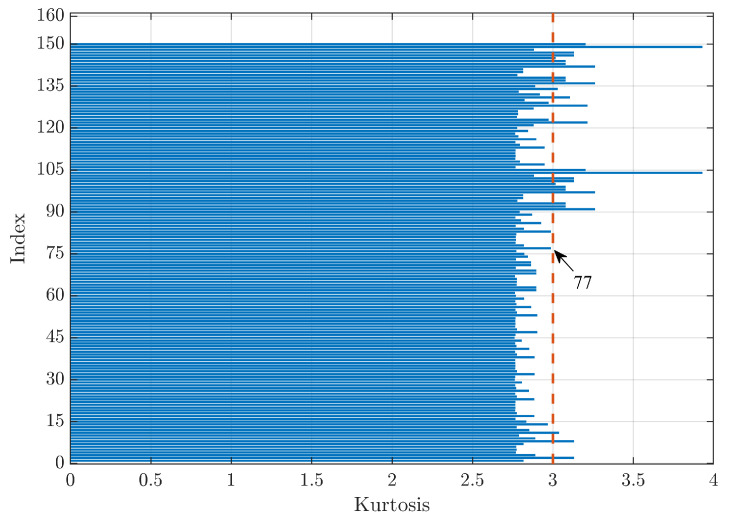
Comparison of the kurtosis values for a healthy bearing. The dashed orange line indicates the wanted kurtosis value in the case of healthy bearings equal to 3.

Figure 12 shows the kurtosis index trends for a signal relative to a healthy bearing. In this case, the screening of the kurtosis parameter corresponds to looking for the method that shows a value as close to 3 as possible, as signals from healthy bearings do not contain any impulsive content due to the interaction between rolling elements and localised defects and, therefore, should present a kurtosis value equal to 3 [2], typical of Gaussian signals. Firstly, the two indices that had peaks close to a kurtosis value of 4 were excluded as they presented a value too large for the analysed case of the healthy bearing signal. Furthermore, it can be noted that there is no regularity among the triads that optimised the parameter in this case. Thus, it was not possible to establish a mother wavelet or thresholding technique that optimises the kurtosis a priori. In the analysed case, the index that minimised the difference between the denoised signal kurtosis value and the searched value equal to 3 was Index 77, which corresponds to the mother wavelet bior6.8, universal as the method of calculating the threshold value, and the improved threshold function. Globally, the difference obtained from the reference value was very low, oscillating between a value of 0.39% up to 8.73%, excluding the two peak values.

Figure 9, Figure 10, Figure 11 and Figure 12 and Table 5 show that the best thresholding techniques for kurtosis screening were the universal threshold and improved threshold function: with these parameters, kurtosis screening was guaranteed except in sporadic cases.

Table 5 shows the optimal parameter groups for each signal analysed. Indices greater than 150 are related to the use of the kurtosis index. In these cases, the group with the thresholding technique that optimised the kurtosis was Index 92.

**Figure 13:**
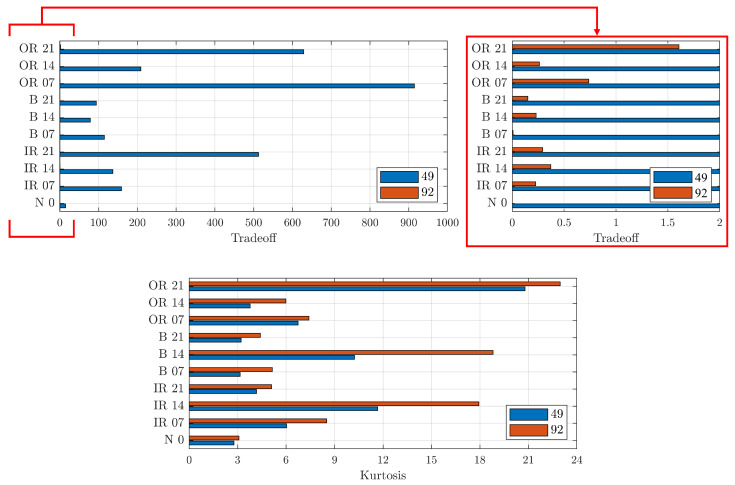
Comparison of TO (**top**) and kurtosis (**bottom**) between Indices 49 and 92 for different defect locations and diameters. On the top-right, zoom of TO graph to highlight values for Index 92. On the *y*-scale, the letters indicate the position of the defect and the numbers indicate the defect diameter size in thousandths of an inch.

Figure 13 presents a comparison between the group of parameters 49 and 92 that optimised the TO and kurtosis parameters, respectively. First of all, it can be seen that the triad 92 did not provide comparable TO values with the maximising 49. On the other hand, for the kurtosis, comparable values were obtained for each signal. Thus, the wavelet parameters with Index 49 was the best for denoising the signals.

In conclusion, Index 49’s parameters should be used if the goal is to further process the signal after denoising. Otherwise, Index 92’s parameters should be used to highlight the presence of a defect, without post-processing the signal.

Kumar et al. [35] tested a denoising method based on the wavelet and modified kurtosis hybrid thresholding rule using the CWRU database, using two different wavelet families, namely Daubechies (Db) and discrete Meyer (dmey). They reported that the mother wavelet Db44 showed the best denoising performance, but with relatively similar results for all other members of both families studied. In the present study, we found that the mother wavelet that resulted in highest kurtosis values for all studied defect locations and sizes was rbior1.1 (if we exclude the analysis based on the kurtosis index technique, Indices 151-160). However, the mother wavelet Db4, a member of the Daubechies family and the first mother wavelet employed in both studies, was the fourth mother wavelet that maximised the kurtosis value, between the 10 analysed in the present study. Moreover, studies in the literature [10] demonstrated how the mother wavelets that result in better performances depend not only on the signals, but also on the evaluation parameter.

### Comparison of Evaluation Parameters on Shaft Speed

A comparison can be made by considering the effects of shaft speed on the evaluation parameters of the denoising process. According to Djebala et al. [7], the highest sampling rate associated with the smallest shock frequency gives a significant kurtosis value of the reconstructed signal. Thus, it is optimal to take the rotation speed as low as possible, and in case that is not possible, the maximal sampling rate is then recommended [7].

**Figure 14:**
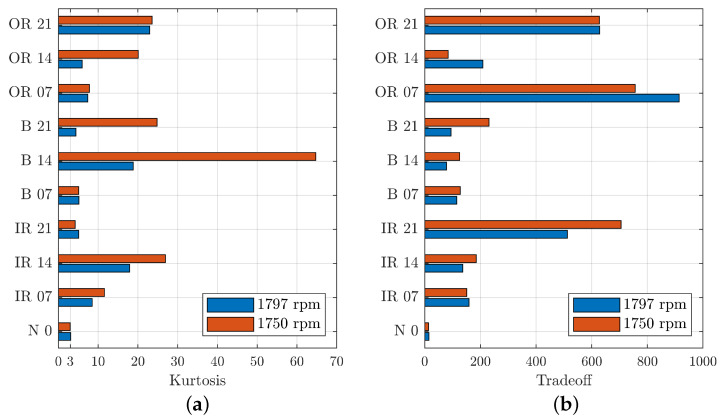
Comparison of kurtosis (**a**) and TO (**b**) between different shaft speeds for different defect locations and diameters. On the *y*-scale, the letters indicate the position of the defect and the numbers indicate the defect diameter size in thousandths of an inch.

Figure 14 illustrates the comparison between the two different shaft speeds. Indeed, Figure 14a shows that, for most signals, there is an optimisation of the kurtosis parameter for lower rotational speeds, in red. In particular, the signal for a healthy bearing has a kurtosis value less than 3 in the case of lower speed, while higher speed results in a kurtosis value greater than 3. For a healthy bearing, the optimisation of this parameter occurs for values closer to 3, which in this case was obtained for the lower speed.

The only cases in which the higher speed ensures an optimised kurtosis value were the cases of the smallest defect located on the rolling elements and the largest defect located on the inner raceway. Thus, the defect within the signal was more evident.

Lower shaft speed favours the denoising of the signal, which can be seen in Figure 14b, where the TO value increases in the case with a lower shaft speed, for most signals. For the analysed signals, an increase in the TO value can be seen, indicating an improvement in the denoising of the signal. This is not respected for signals relating to defective bearings on the outer ring (OR), for which there was a better TO value for higher shaft speed.

In conclusion, the decrease in speed led indicatively to an increase in the evaluation parameters, keeping the trends constant.

## 4. Classification of Processed Signals

Classification gives an understanding of the origin and, in some cases, the extent of the defect, so that maintenance can be planned in the best possible way.

Table 6 presents the set of extracted features in the time domain that were provided as the input to the classification algorithm. As a first step, the features were selected through the Fisher score and the Pareto rule, as explained in [17]. The second step was PCA, which provides a hierarchical coordinate system for data representation. The geometry of the resulting coordinate system is determined by the PCs that have the highest correlation with the measurements.

**Figure 15:**
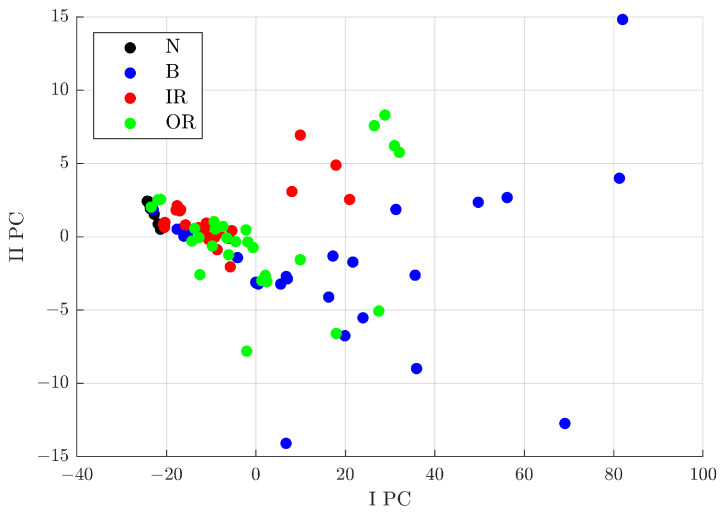
Distribution in the I PC–II PC plane of the analysed signals with respect to the characteristics selected by the Pareto method out of 11, calculated for 4 defect classes after denoising with Group 49.

Signals from healthy and defective bearings on the inner raceway, outer raceway, and rolling elements were considered for a total of 113 signals. Another analysis was carried out only on the defect classes, excluding signals from healthy bearings, for a total of 105 signals analysed. In the first case, the Fisher–Pareto method extracted six features, whereas in the second one, five. Although, in both cases, the features with the highest Fisher score were the crest factor, impulse factor, and clearance factor, which are directly proportional to the peak value. Therefore, these features are closely related to each other. Figure 15 shows the results obtained by the classification algorithm on the signals processed through the denoising process with the parameters related to Index 49, considering all defect classes, on the left, and all the ones excluding the signals from healthy rolling bearings. The axes correspond to the first two PCs extracted by the algorithm. The graph shows the zones of interest for each defect class, where the state class of a bearing can be established with a probability, depending on the location of the signal on this plane. In particular, Figure 15 shows different areas, as:

- IPC<−20: in this area, signals from a healthy rolling bearing can be found;
−20<IPC<0: the area with a higher probability of signals from a rolling bearing with a defect on the inner or outer raceway;
- IPC>0: the area with a higher probability of signals from a rolling bearing with a defect on the rolling elements.

In these cases, the PCA process provided a distribution of signals that cannot be obtained only considering the features extracted in the time domain. Indeed, the signal distribution on a plain formed by the first two features with the higher Fisher score does not allow defining an area of interest for any defect class.

**Figure 16:**
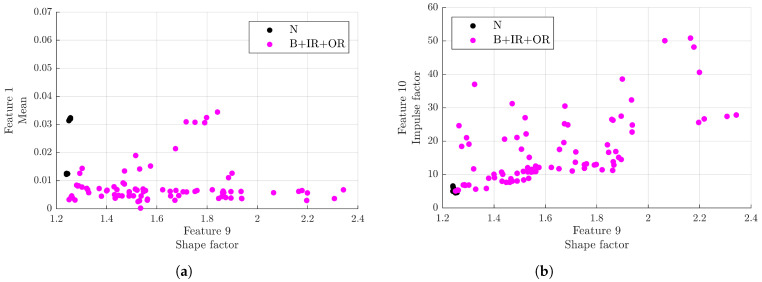
Distribution of signals with respect to the time domain features selected by the Fisher–Pareto algorithm: (**a**) plane formed by the first (shape factor) and second (mean) selected features; (**b**) plane formed by the first (shape factor) and third (impulse factor) selected features.

To facilitate the classification, a comparison between signals from healthy bearings and from rolling bearings with localised defects is shown in Figure 16. In this case, the best distributions were obtained considering the planes formed by the first three features with the highest Fisher score, in order, the shape factor (F9), mean (F1), and impulse factor (F10). In particular, observing the two planes in Figure 16, distinct zones generated by healthy and damaged bearing signals can be observed:

- F9≃1.25∩0.01<F1<0.04: the area with a higher probability of a healthy rolling bearing;
- F9>1.3∩F10>6.5: the area with a higher probability of signals from a rolling bearing with a localised defect.

**Figure 17:**
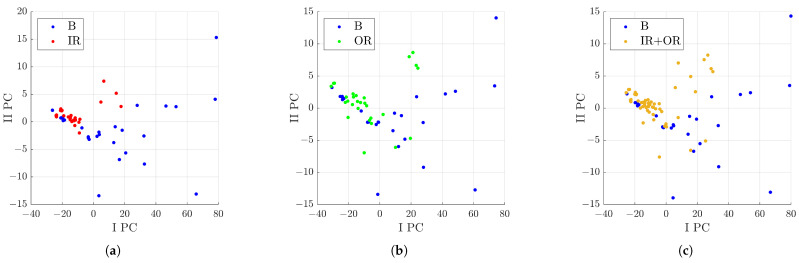
Distribution in the I PC–II PC plane of the analysed signals with respect to the characteristics selected by the Pareto method out of 11 after denoising with Group 49: (**a**) comparison between B and IR classes; (**b**) comparison between B and OR classes; (**c**) comparison between B and IR+OR classes.

Moreover, other analyses were carried out to define the distribution of the signals considering the subgroups of the defect classes. Signals from rolling bearings with a defect on the rolling element were compared with the defect class IR, then with OR, and finally, with signals from both IR and OR classes. For all the comparisons, the feature with the highest Fisher score was the crest factor. In particular, a higher value of the crest factor indicates signals from the B class; conversely, lower values are for signals from the IR and OR classes. Figure 17 shows the distribution of the signals in the various cases. The graph in (c) can be considered similar to the sum of the (a) and (b) ones. In particular, in Figure 17c, some interesting area for the defect classes can be evidenced, as:

- IPC<0∩−2.5<IIPC<2.5: the area with a higher probability to find signals of a rolling bearing with a defect on the inner or outer raceway;
- IPC>0∩F8>10: the area with a higher probability of signals from a rolling bearing with a defect on the rolling elements.

**Figure 18:**
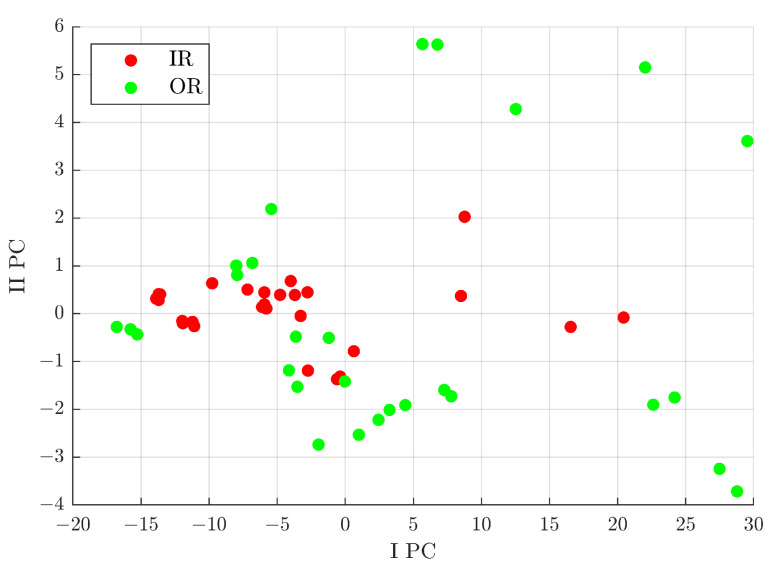
Distribution in the I PC–II PC plane of the analysed signals, from a rolling bearing with a defect on the inner raceway (IR) and on the outer one (OR), with respect to the characteristics selected by the Pareto method out of 11 after denoising with Group 49.

These analyses allow distinguishing signals of the B class from the IR+OR classes, but with this subgroup of classes it is not possible to define the difference of IR from OR. Therefore, the next analysis considered was focused on the comparison between signals from rolling bearings with defects on the inner raceway to those with defects on the outer one. From the pre-selection process, five features were extracted; in particular, the first two with the highest fisher score were, in order, the clearance factor and impulse factor. Figure 18 shows the distribution after the PCA process. In particular, in Figure 18, some interesting areas for the defect classes can be evidenced:

- IPC<0∩IIPC>0: the area with a higher probability of signals from a rolling bearing with a defect on the inner raceway;
- IIPC<0: the area with a higher probability of signals from a rolling bearing with a defect on the outer raceway.

**Figure 19:**
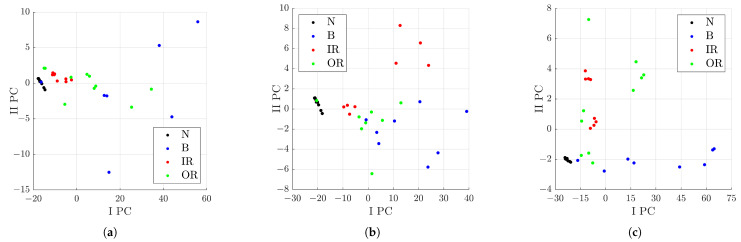
Distribution in the I PC–II PC plane of the analysed signals with respect to the characteristics selected by the Pareto method out of 11 after denoising with Group 49; (**a**) comparison of a defect dimension of 0.007 in; (**b**) comparison of a defect dimension of 0.014 in; (**c**) comparison of a defect dimension of 0.021 in.

The last analysis was the application of the classification algorithm to all defect classes for defects of the same dimension, which is shown in Figure 19. From the graphs in (a) to (c), with the increasing of the dimension of the defect, the areas of interest of each defect class are more distinct. Therefore, a better classification can be obtained if the defect has a larger size.

## 5. Conclusions

In this paper, a method for selecting the optimal parameters for denoising signals using the DWT was presented, considering in particular the mother wavelet, threshold rule, and threshold function.

The analysis carried out on the CWRU database revealed that the set of variables formed by the dmey, rigrSURE threshold, and hard threshold function (related to Index 49) provided the best denoising evaluated through the TO parameter.

On the other hand, using the kurtosis as an evaluation parameter, it was obtained that the rbior1.1, universal threshold, and improved threshold function (related to Index 92) were the best DWT parameters. The work presented resulted in:

- The identification of the parameter set that maximised the tradeoff value considering all the localised defect typologies: the highlighted parameter set was identified by Index 49 and presented the mother wavelet Dmey, rigrSURE threshold evaluation method, and hard threshold function;
- The identification of the parameter set that maximised the kurtosis value of the denoised signals considering all the localised defect typologies: the identified set was Index 92, which employed the mother wavelet rbior1.1, universal threshold evaluation method, and improved threshold function;
- The selection of Index 49’s parameter set as the best parameter combination that resulted in more effective denoising between 49 and 92;
- The classification of the denoised signals using Index 49’s parameter set with the industry sector algorithm based on the Fisher–Pareto method and PCA.

A possible application of this method can be to identify the parameters that optimise the two evaluation methods and to use the one obtained from the kurtosis to identify the presence or absence of the defect, after which denoising with the group obtained by TO can be carried out, then using the signal and its components in the following analyses.

In addition, using the analysed signals and the described dataset, initial training of the classification algorithm was possible to extract the bearing condition from the signal. It should be noted that the results obtained in the present work were related to signals from the CWRU database; the results will be extended in future works to achieve the optimal parameters for more generalised sets of bearing signals, by including signals from different test benches.

## Figures and Tables

**Table 1:** Mother wavelet used in the screening algorithm.

Mother Wavelet	Abbreviation	Literary Sources
Daubechies 4	db4	[10,16]
Daubechies 44	db44	[10,11,17]
Daubechies 45	db45	[10,11]
Discrete Meyer	dmey	[9,10]
Biorthogonal 3.1	bior3.1	[10,11]
Biorthogonal 6.8	bior6.8	[9,10]
Reverse biorthogonal 1.1	rbior1.1	[9,10]
Coiflet 5	coif5	[9,10]
Symlet 4	sym4	[10,16]
Haar	haar	[10,16]

**Table 2:** Parameters required to maximise the TO value for rolling bearings’ signals with localised defects with varying locations, but the same diameter, equal to 0.014 in.

Defect Location	Index	Mother Wavelet	Threshold Rule	Threshold Function
IR	19	db44	rigrSURE	Hard threshold
B	19	db44	rigrSURE	Hard threshold
OR	58	dmey	Penalised method	Hard threshold

**Table 3:** Parameters required to maximise the sum of TO values for every defect location at varying defect diameters.

Diameter (in)	Index	Mother Wavelet	Threshold Rule	Threshold Function
0.007	49	dmey	rigrSURE	Hard threshold
0.014	49	dmey	rigrSURE	Hard threshold
0.021	49	dmey	rigrSURE	Hard threshold

**Table 4:** Summary of denoising parameters that maximise TO for different defect locations and diameters.

Defect Location	Diameter (in)	Index	Mother Wavelet	Threshold Rule	Threshold Function
N	–	34	db45	rigrSURE	Hard threshold
IR	0.007	49	dmey	rigrSURE	Hard threshold
IR	0.014	19	db44	rigrSURE	Hard threshold
IR	0.021	49	dmey	rigrSURE	Hard threshold
B	0.007	49	dmey	rigrSURE	Hard threshold
B	0.014	19	db44	rigrSURE	Hard threshold
B	0.021	19	db44	rigrSURE	Hard threshold
OR	0.007	49	dmey	rigrSURE	Hard threshold
OR	0.014	58	dmey	Penalised method	Hard threshold
OR	0.021	49	dmey	rigrSURE	Hard threshold

**Table 5:** Summary of denoising parameters that optimise kurtosis for different defect locations and diameters.

Defect Location	Diameter (in)	Index	Mother Wavelet	Threshold Rule	Threshold Function
N	–	77	bior6.8	Universal	Improved threshold
IR	0.007	92	rbior1.1	Universal	Improved threshold
IR	0.014	151	db4	–	–
IR	0.021	154	dmey	–	–
B	0.007	92	rbior1.1	Universal	Improved threshold
B	0.014	92	rbior1.1	Universal	Improved threshold
B	0.021	157	rbior1.1	–	–
OR	0.007	154	dmey	–	–
OR	0.014	92	rbior1.1	Universal	Improved threshold
OR	0.021	159	sym4	–	–

**Table 6:** List of time domain features extracted from the signals for the input matrix of the classification algorithm.

No.	Feature
1	Mean
2	Peak to peak value
3	Peak value
4	Standard deviation
5	Skewness
6	Kurtosis
7	Root mean square
8	Crest factor
9	Shape factor
10	Impulse factor
11	Clearance factor

## Data Availability

Publicly available datasets were analyzed in this study. This data can be found here: https://engineering.case.edu/bearingdatacenter/download-data-file (accessed on 6 November 2022).

## Appendix A

See Table A1.

**Table A1:** Correspondence between indices and groups of DWT parameters: mother wavelet, threshold rule, and threshold function.

Index	Mother Wavelet	Threshold Rule	Threshold Function
1	db4	Universal	Hard threshold
2	db4	Universal	Improved threshold
3	db4	Universal	Mixed threshold
4	db4	rigrSURE	Hard threshold
5	db4	rigrSURE	Improved threshold
6	db4	rigrSURE	Mixed threshold
7	db4	heuresure	Hard threshold
8	db4	heuresure	Improved threshold
9	db4	heuresure	Mixed threshold
10	db4	minimax	Hard threshold
11	db4	minimax	Improved threshold
12	db4	minimax	Mixed threshold
13	db4	Penalised method	Hard threshold
14	db4	Penalised method	Improved threshold
15	db4	Penalised method	Mixed threshold
16	db44	Universal	Hard threshold
17	db44	Universal	Improved threshold
18	db44	Universal	Mixed threshold
19	db44	rigrSURE	Hard threshold
20	db44	rigrSURE	Improved threshold
21	db44	rigrSURE	Mixed threshold
22	db44	heuresure	Hard threshold
23	db44	heuresure	Improved threshold
24	db44	heuresure	Mixed threshold
25	db44	minimax	Hard threshold
26	db44	minimax	Improved threshold
27	db44	minimax	Mixed threshold
28	db44	Penalised method	Hard threshold
29	db44	Penalised method	Improved threshold
30	db44	Penalised method	Mixed threshold
31	db45	Universal	Hard threshold
32	db45	Universal	Improved threshold
33	db45	Universal	Mixed threshold
34	db45	rigrSURE	Hard threshold
35	db45	rigrSURE	Improved threshold
36	db45	rigrSURE	Mixed threshold
37	db45	heuresure	Hard threshold
38	db45	heuresure	Improved threshold
39	db45	heuresure	Mixed threshold
40	db45	minimax	Hard threshold
41	db45	minimax	Improved threshold
42	db45	minimax	Mixed threshold
43	db45	Penalised method	Hard threshold
44	db45	Penalised method	Improved threshold
45	db45	Penalised method	Mixed threshold
46	dmey	Universal	Hard threshold
47	dmey	Universal	Improved threshold
48	dmey	Universal	Mixed threshold
49	dmey	rigrSURE	Hard threshold
50	dmey	rigrSURE	Improved threshold
51	dmey	rigrSURE	Mixed threshold
52	dmey	heuresure	Hard threshold
53	dmey	heuresure	Improved threshold
54	dmey	heuresure	Mixed threshold
55	dmey	minimax	Hard threshold
56	dmey	minimax	Improved threshold
57	dmey	minimax	Mixed threshold
58	dmey	Penalised method	Hard threshold
59	dmey	Penalised method	Improved threshold
60	dmey	Penalised method	Mixed threshold
61	bior3.1	Universal	Hard threshold
62	bior3.1	Universal	Improved threshold
63	bior3.1	Universal	Mixed threshold
64	bior3.1	rigrSURE	Hard threshold
65	bior3.1	rigrSURE	Improved threshold
66	bior3.1	rigrSURE	Mixed threshold
67	bior3.1	heuresure	Hard threshold
68	bior3.1	heuresure	Improved threshold
69	bior3.1	heuresure	Mixed threshold
70	bior3.1	minimax	Hard threshold
71	bior3.1	minimax	Improved threshold
72	bior3.1	minimax	Mixed threshold
73	bior3.1	Penalised method	Hard threshold
74	bior3.1	Penalised method	Improved threshold
75	bior3.1	Penalised method	Mixed threshold
76	bior6.8	Universal	Hard threshold
77	bior6.8	Universal	Improved threshold
78	bior6.8	Universal	Mixed threshold
79	bior6.8	rigrSURE	Hard threshold
80	bior6.8	rigrSURE	Improved threshold
81	bior6.8	rigrSURE	Mixed threshold
82	bior6.8	heuresure	Hard threshold
83	bior6.8	heuresure	Improved threshold
84	bior6.8	heuresure	Mixed threshold
85	bior6.8	minimax	Hard threshold
86	bior6.8	minimax	Improved threshold
87	bior6.8	minimax	Mixed threshold
88	bior6.8	Penalised method	Hard threshold
89	bior6.8	Penalised method	Improved threshold
90	bior6.8	Penalised method	Mixed threshold
91	rbio1.1	Universal	Hard threshold
92	rbio1.1	Universal	Improved threshold
93	rbio1.1	Universal	Mixed threshold
94	rbio1.1	rigrSURE	Hard threshold
95	rbio1.1	rigrSURE	Improved threshold
96	rbio1.1	rigrSURE	Mixed threshold
97	rbio1.1	heuresure	Hard threshold
98	rbio1.1	heuresure	Improved threshold
99	rbio1.1	heuresure	Mixed threshold
100	rbio1.1	minimax	Hard threshold
101	rbio1.1	minimax	Improved threshold
102	rbio1.1	minimax	Mixed threshold
103	rbio1.1	Penalised method	Hard threshold
104	rbio1.1	Penalised method	Improved threshold
105	rbio1.1	Penalised method	Mixed threshold
106	coif5	Universal	Hard threshold
107	coif5	Universal	Improved threshold
108	coif5	Universal	Mixed threshold
109	coif5	rigrSURE	Hard threshold
110	coif5	rigrSURE	Improved threshold
111	coif5	rigrSURE	Mixed threshold
112	coif5	heuresure	Hard threshold
113	coif5	heuresure	Improved threshold
114	coif5	heuresure	Mixed threshold
115	coif5	minimax	Hard threshold
116	coif5	minimax	Improved threshold
117	coif5	minimax	Mixed threshold
118	coif5	Penalised method	Hard threshold
119	coif5	Penalised method	Improved threshold
120	coif5	Penalised method	Mixed threshold
121	sym4	Universal	Hard threshold
122	sym4	Universal	Improved threshold
123	sym4	Universal	Mixed threshold
124	sym4	rigrSURE	Hard threshold
125	sym4	rigrSURE	Improved threshold
126	sym4	rigrSURE	Mixed threshold
127	sym4	heuresure	Hard threshold
128	sym4	heuresure	Improved threshold
129	sym4	heuresure	Mixed threshold
130	sym4	minimax	Hard threshold
131	sym4	minimax	Improved threshold
132	sym4	minimax	Mixed threshold
133	sym4	Penalised method	Hard threshold
134	sym4	Penalised method	Improved threshold
135	sym4	Penalised method	Mixed threshold
136	haar	Universal	Hard threshold
137	haar	Universal	Improved threshold
138	haar	Universal	Mixed threshold
139	haar	rigrSURE	Hard threshold
140	haar	rigrSURE	Improved threshold
141	haar	rigrSURE	Mixed threshold
142	haar	heuresure	Hard threshold
143	haar	heuresure	Improved threshold
144	haar	heuresure	Mixed threshold
145	haar	minimax	Hard threshold
146	haar	minimax	Improved threshold
147	haar	minimax	Mixed threshold
148	haar	Penalised method	Hard threshold
149	haar	Penalised method	Improved threshold
150	haar	Penalised method	Mixed threshold
151	db4	–	kurtosis_index
152	db44	–	kurtosis_index
153	db45	–	kurtosis_index
154	dmey	–	kurtosis_index
155	bior3.1	–	kurtosis_index
156	bior6.8	–	kurtosis_index
157	rbio1.1	–	kurtosis_index
158	coif5	–	kurtosis_index
159	sym4	–	kurtosis_index
160	haar	–	kurtosis_index
